# International flight-related transmission of pandemic influenza A(H1N1)pdm09: an historical cohort study of the first identified cases in the United Kingdom

**DOI:** 10.1111/irv.12181

**Published:** 2013-11-07

**Authors:** Nicholas Young, Richard Pebody, Gillian Smith, Babatunde Olowokure, Giri Shankar, Katja Hoschler, Monica Galiano, Helen Green, Anders Wallensten, Angela Hogan, Isabel Oliver

**Affiliations:** a Health Protection AgencyBristol, UK; b Health Protection Agency, Centre for InfectionsColindale, UK; c Health Protection AgencyBirmingham, UK; d Health Protection AgencyThetford, UK; e Swedish Institute for Communicable Disease ControlStockholm, Sweden

**Keywords:** Aircraft, influenza, pandemics, prevention and control, transmission, travel

## Abstract

**Background:**

Transporting over two billion passengers per year, global airline travel has the potential to spread emerging infectious diseases, both via transportation of infectious cases and through in-flight transmission. Current World Health Organization (WHO) guidance recommends contact tracing of passengers seated within two rows of a case of influenza during air travel.

**Objectives:**

The objectives of this study were to describe flight-related transmission of influenza A(H1N1)pdm09 during a commercial flight carrying the first cases reported in the United Kingdom and to test the specific hypothesis that passengers seated within two rows of an infectious case are at greater risk of infection.

**Methods:**

An historical cohort study, supplemented by contact tracing, enhanced surveillance data and laboratory testing, was used to establish a case status for passengers on board the flight.

**Results:**

Data were available for 239 of 278 (86·0%) of passengers on the flight, of whom six were considered infectious in-flight and one immune. The attack rate (AR) was 10 of 232 (4·3%; 95% CI 1·7–6·9%). There was no evidence that the AR for those seated within two rows of an infectious case was different from those who were not (relative risk 0·9; 95% CI 0·2–3·1; *P* = 1·00). Laboratory testing using PCR and/or serology, available for 118 of 239 (49·4%) of the passengers, was largely consistent with clinically defined case status.

**Conclusions:**

This study of A(H1N1)pdm09 does not support current WHO guidance regarding the contact tracing of passengers seated within two rows of an infectious case of influenza during air travel.

## Introduction

An emergent novel swine-origin influenza A(H1N1)pdm09 virus was first identified in humans on 15th April 2009; on 25th April 2009, the World Health Organization declared the outbreak a ‘public health emergency of international concern’.^1^ The subsequent influenza pandemic spread from Mexico to at least 214 countries with over 18 000 laboratory-confirmed deaths worldwide, with the true burden of disease many times greater.^2^,^3^

With over 2 billion passengers per year, global airline travel represents an important vehicle for the spread of emerging infectious diseases such as pandemic influenza, both through the transportation of infected passengers and through the potential for in-flight transmission of disease. Previous studies have described the spread of influenza during air travel,^4^,^5^ including five reports of transmission of influenza A(H1N1)pdm09 on commercial flights.^6^–^10^ Current World Health Organization (WHO) technical advice for case management of influenza infection in-flight includes specific measures to reduce transmission and recommends the completion of passenger locator cards to facilitate subsequent contact tracing of passengers seated within two rows of an infected case.^11^

The aims of this study are to describe the in-flight transmission of influenza A(H1N1)pdm09 during a commercial flight carrying the first cases reported in the United Kingdom and to test the specific hypothesis that passengers seated within two rows of an infected case are at greater risk of infection. Knowledge of in-flight transmission dynamics of influenza A(H1N1)pdm09 will inform public health policy regarding advice on dealing with potentially infectious passengers and the role, if any, of contact tracing.

## Methods

On 27th April 2009, a couple returning by air travel from vacation in Mexico became the first case of influenza A(H1N1)pdm09 to be reported to the public health agencies in the United Kingdom (UK). The couple travelled on a Boeing 767–300 that departed Cancun, Mexico, at 0000 GMT on 21st April 2009, and arrived in Birmingham, UK, 9·5 hours later. This cohort study, supplemented by multiple overlapping data sources, examines possible transmission dynamics between passengers travelling on board the same commercial flight.

### Data sources

The data sources used were an historical cohort study using a telephone questionnaire administered following the flight; initial contact tracing of passengers seated within two rows of the first identified case and enhanced surveillance data collected by the Health Protection Agency (HPA) at the time of the pandemic.

#### Historical cohort study

A flight manifest was obtained to identify a study cohort, the sampling frame of which comprised all passengers travelling on the flight. The HPA attempted to contact all passengers to request completion of a telephone-administered questionnaire to investigate demographics, location of stay and activities in Mexico, travelling group links and symptoms. Passengers were asked which seat they sat in, as some did not take up their allocated seat. Eight public health officials conducted the interviews between 9th June 2009 and 5th August 2009. Passengers unable to be contacted by telephone after three attempts were sent an invitation to participate by post, but there were no further responses to this. All passengers contacted were invited to attend for serological testing (see section Laboratory testing).

#### Contact tracing

The West Midlands HPA regional epidemiology unit attempted to contact those seated within two rows of the first two reported cases on the flight, to assess case status and the individual passenger's need for antiviral medication. All contact tracing was performed between 29th April 2009 and 1st May 2009, by two officials.

#### HPA enhanced surveillance data

Data linkage was used to cross-match the flight manifest with HPA information collected at the time of the pandemic using the first few hundred (FF100) and Data Mart surveillance systems.^12^ The FF100 surveillance system collected detailed demographic and clinical data via interviews and review of clinical records from the first 392 reported laboratory cases of influenza A(H1N1)pdm09 in the UK.

The Data Mart project was developed during the pandemic as an automated surveillance tool to collect H1N1 testing information from UK laboratories, in an effort to improve timeliness and completion of reporting. Data Mart data fields include clinical information and the results of PCR testing.

### Definition of Influenza-like illness and case status of cohort subjects

The USA Centre for Disease Control and Prevention (CDC) clinical definition of influenza-like illness (ILI) was used, defined as fever (self-reported in this study), with at least one symptom of sore throat or cough.

To establish the case status of passengers, an incubation time for influenza A(H1N1)pdm09 of 0–6 days was used, with a period of communicability from 1 day pre- to 1 day post-symptom resolution.^13^ The case status of subjects in the cohort was defined as infectious in-flight; immune in-flight; infection acquired in-flight; non-case; and unknown.^9^

#### Infectious in-flight (*Infectious*)

Any passenger with symptoms compatible with ILI and onset prior to the day of the flight, with recovery of symptoms no earlier than the day before the flight.

#### Immune in-flight (*Immune)*

Any passenger with a history of symptoms compatible with ILI, but who had recovered at least 2 days before the flight.

#### Infection acquired in-flight (*Infected in-flight*)

Any passenger with a history of symptoms compatible with ILI that began 0–6 days after the day of the flight.

#### Non-case (*non-case)*

A passenger with clinical data who did not have symptoms consistent with ILI or a date of onset more than 6 days post-flight.

### Statistical analysis

All data analysis was performed using Stata 12.0 (Stata Corp., College Station, TX, USA). As the data sources were overlapping, they were considered to be a hierarchy, with the cohort study considered the most complete and reliable for clinical data, followed by contact tracing and then laboratory surveillance data. Initial descriptive analysis was performed, mapping the flight seating plan, followed by calculation of the attack rate and testing the primary hypothesis using Fisher's exact test.

### Laboratory testing

Combined nose and throat swab specimens were analysed by RT-PCR for detection of influenza A (H1N1)pdm09 virus as described previously.^14^ Blood samples for serological testing were collected by study nurses and shipped to the HPA laboratory (Colindale, London, UK), where serum was separated and stored at −20°C until analysed. Antibody responses were detected by haemagglutination inhibition (HI) according to the standard methods.^15^ Sera and specific positive and negative control sera were treated with receptor-destroying enzyme (RDE II) (Denka Seiken Co., Ltd., Tokyo, Japan) according to manufacturer's protocol to remove non-specific inhibitors. HI antibody titres were determined in duplicate for each serum using four haemagglutinating units (HA units) of either NIBRG121 or NIBRG122 (reverse genetics viruses with PR8 backbone and NA and HA components of the A/California/7/2009 and A/England/195/2009 viruses, respectively) and a suspension of Turkey red blood cells. Titres were expressed as the reciprocal of the highest dilution of serum completely inhibiting the haemagglutination reaction. Single samples with titres ≥1:32 by HI were considered seropositive, suggestive of recent infection with influenza A(H1N1)pdm09.

## Results

Table 1 demonstrates the results of serological and PCR testing by case status for passengers on the flight. In total, 30 passengers had PCR testing with seven positive for influenza A(H1N1)pdm09. Serology testing was available for 96 of 239 (40·2%) passengers (two *infected in-flight* and 94 *non-cases*).

**Table 1:** PCR and serology results by case status (*n* = 239)

Clinical case status	Underwent PCR testing (%)	PCR result		Underwent serological testing (%)	Serology result	
	
		Positive (%)	Negative		Positive (%)	Negative
*Non-case*	22/222 (9·9)	2 (9·1)	20	94/222 (42·3)	11 (11·7)	83
*Infectious*	4/6 (66·7)	1 (25·0)	3	0/6 (0·0)	n/a	n/a
*Immune*	0/1 (0)	n/a	n/a	0/1 (0·0)	n/a	n/a
*Infected in-flight*	4/10 (40·0)	4 (100)	0	2/10 (20·0)	1 (50·0)	1

**Figure 1:**
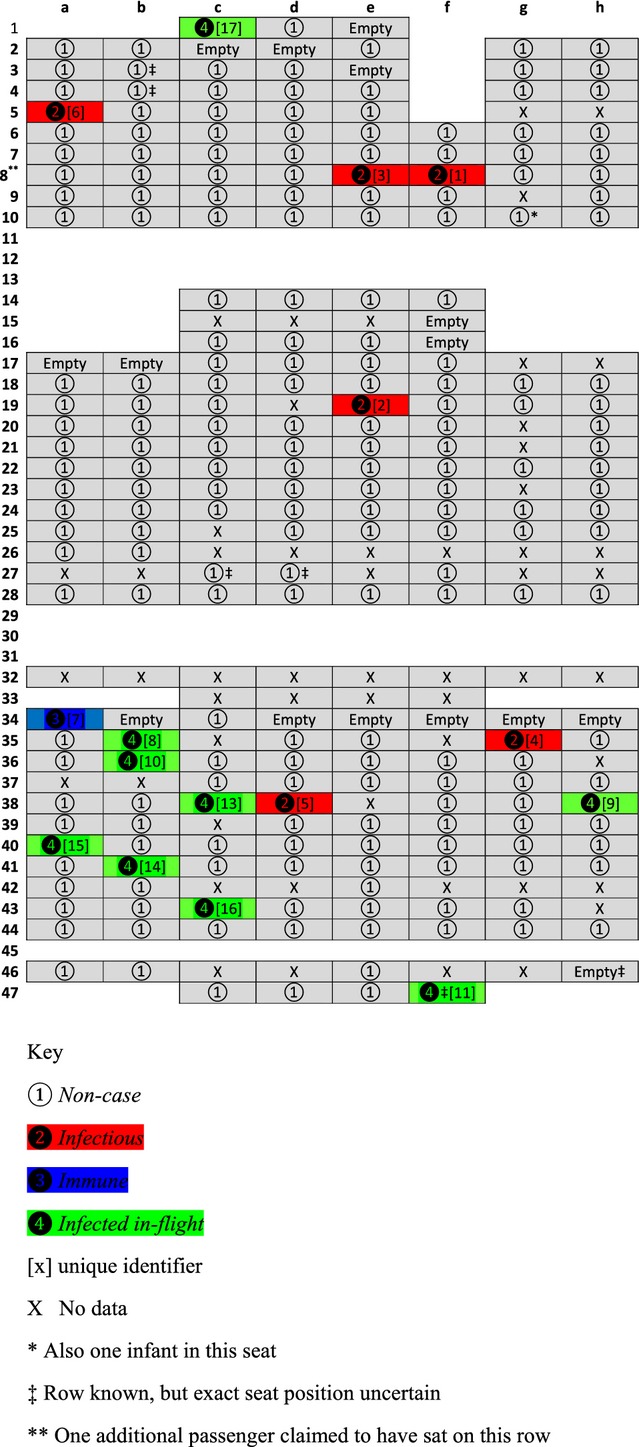
Schematic diagram of flight seating plan with case status of passengers.

Clinical data sufficient to establish a case definition were available for 239 of the 278 (86·0%) passengers who travelled on the flight of which 224 were part of the cohort study; an additional 12 were added through contact tracing. Enhanced surveillance data were available for 17 passengers, but only contributed information regarding an additional three cases. Ten passengers were defined clinically as *infected in-flight*, leading to an overall attack rate of 10 of 232 at risk (4·3%; 95% CI 1·7–6·9%). Of the ten passengers defined as *infected in-flight*, four were tested using PCR, and of these, 4 of 4 (100%) were positive, one PCR positive case was also serology positive, and a further passenger who did not undergo PCR testing was serology negative. Six passengers were defined as *infectious*, of whom four underwent PCR testing, with one-fourth (25%) testing positive; no *infectious* cases underwent serological testing. One passenger was defined as *immune* during the flight and underwent no laboratory testing. Figure 1 demonstrates the seating plan for the flight; eight *infected in-flight* cases were seated in the rear section of the plane, with one seated at the front. The seat location of one *infected in-flight* case was unknown. *Infectious* cases were seated in all sections of the flight. There was no evidence that passengers seated within two rows of an *infectious* case were at an increased risk of infection compared with those who were not (relative risk 0·9; 95% CI 0·2–3·1), with the attack rates similar for both groups (5 of 131, 3·8%; 95% CI 0·5%–7·1% versus 4 of 89, 4·5%; 95% CI 0·1–8·9%; *P* = 1·00); seating data were unavailable for 12 passengers at risk.

**Figure 2:**
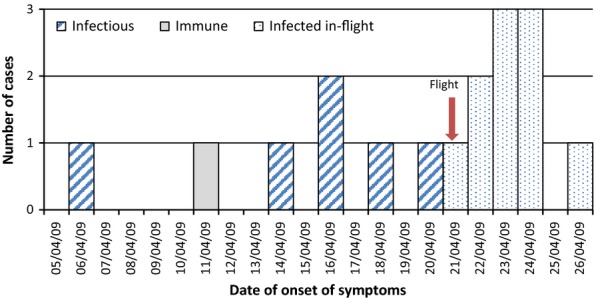
Epidemic Curve by case status (*n* = 17).

Figure 2 demonstrates the epidemic curve for those with ILI, and all cases *infected in-flight* occurred between 0 and 6 days post-flight with 9 of 10 occurring within 3 days.

The median age of all passengers with ILI was 16 years [mean 26 (SD 17), range 6–62 (IQR 15–46)], compared with 37 years [mean 33 (SD 18); range 0–76 (IQR 12–43); difference in mean between groups 7 years] for those without. A similar proportion of passengers with or without ILI were male (48·2% versus 47·1%).

### Other potential modes of contact

Apart from the first two UK-reported cases, *infectious* case 5 and *infected in-flight* case 13 (Table 2), no other passengers *infected in-flight* had travelled in Mexico with *infectious* cases. Further information gathered for the 224 participants in the cohort study did not reveal any specific links with resorts in Mexico, with only three of eight (two unknown) of the *infected in-flight* passengers staying at resorts where those considered *infectious* had been on vacation; *infected in-flight* cases 9 and 10 stayed at the same resort as *infectious* case 2; *infected in-flight* case 16 stayed at the same resort as *infectious* case 4. All passengers travelled to the airport on tour buses, an estimated journey time of 30–120 minutes depending on starting location. Where information was available, passengers with ILI stayed at either Cancun (two *infectious*, five *infected in-flight*), Riviera Maya (three *infectious*, one *immune*, two *infected in-flight*) or Akumal (one *infected in-flight*). Cancun is north of Cancun airport, whereas Riviera Maya and Akumal are south; therefore, it seems unlikely that these passengers travelled together to the airport. No apparent links between those with ILI could be attributed to day trips and visits while in Mexico.

**Table 2:** Unique identifier, dates of onset and recovery of ILI, and resort stayed at during week prior to flight, by case status (Addendum to Figure 1)

Case status	Unique id	Date of onset of ILI	Resort	Town/City	Date of recovery from ILI
*Infectious*	1	06/04/2009	Iberostar Tucan	Riviera Maya	27/04/2009
	2	14/04/2009	Moon Palace	Cancun	05/05/2009
	3	16/04/2009	Iberostar Tucan	Riviera Maya	missing
	4	16/04/2009	Sandos_Caracol	Riviera Maya	02/05/2009
	5*	18/04/2009	n/a***	n/a***	n/a***
	6	20/04/2009	Riu Cancun	Cancun	28/04/2009
*Immune*	7	11/04/2009	Sandos Caracol	Riviera Maya	15/04/2009
*Infected*	8	21/04/2009	Royal Cancun	Cancun	28/04/2009
*In-flight*	9	22/04/2009	Moon Palace	Cancun	29/04/2009
	10	22/04/2009	Moon Palace	Cancun	25/04/2009
	11	23/04/2009	Riu Caribe	Cancun	missing
	12**	23/04/2009	Catalonia	Riviera Maya	30/04/2009
	13*	23/04/2009	n/a***	n/a***	n/a***
	14	24/04/2009	Riu Palace las Americas	Cancun	28/04/2009
	15	24/04/2009	n/a***	n/a***	n/a***
	16	24/04/2009	Sandos Caracol	Riviera Maya	28/04/2009
	17	26/04/2009	Hacienda	Akumal	08/05/2009

* Travelled together.

** seat position unknown.

*** Data unavailable as case not in historical cohort study.

There was no association between time spent in the departure lounge and being *infected in-flight* (OR trend 1·1, 95% CI 0·4–3·0; *P* = 0·88). Four of eight (50%; two missing data) of the *infected in-flight* cases spent more than two hours in the departure lounge compared with 114 of 208 (54·8%; 14 missing data) of *non-cases*.

Participants in the cohort study were asked about toilet use on board the flight, one *infectious* case used the front right toilet, with information not available for the remaining five. The relative risk of infection by exposure to the toilet used by *infectious* cases could therefore not be calculated. Six of ten cases *infected in-flight* provided information on toilet use, using three different toilets, with two using the front right toilet. Data regarding movement in-flight were available for five of the six *infectious* passengers, with one stating they spent 1–2 hours away from their seat and a further two away from their seat for over two hours, whereas all *infected in-flight* cases spent <1 hour away from their allocated seat.

## Discussion

### Main findings

This study estimates the overall attack rate (AR) for influenza A(H1N1)pdm09 on a nine-hour commercial flight with six potentially infectious passengers on board to be 4·3%, but did not demonstrate an increased risk to those seated within two rows of an infectious case.

The AR in our present study is largely consistent with the published data from four previous reports that have suggested transmission of influenza A(H1N1)pdm09 during long-haul modern commercial air travel; 0·5% for all sections of a 13-hour flight with a single infectious passenger^8^; 4·7% in a single section of a 15-hour flight with a single infectious passenger^10^; 1·9% in a single section containing 12 infective passengers on a 13-hour flight^9^; 2·4% estimated using pooled data from four flights of between 1·5- and 2·5-hour durations.^16^ Han *et al*.^7^ reported a single case (AR 1·1%) infected on a 45-minute flight containing two infectious cases.

Our study does not demonstrate an increased risk of becoming infected with influenza A(H1N1)pdm09 in those seated within two rows of an infectious case compared with those who were not. Two separate reports of single cases infected in-flight demonstrated that they sat two and six rows away from the known infectious case.^7^,^8^ Ooi *et al*.^10^demonstrated that contact tracing of passengers within two rows of an infectious case would have only detected two of five of cases newly infected on a long-haul flight with a single known infectious passenger. The attack rate for those seated within two rows of any of 12 infectious cases on a flight from Los Angeles to Auckland was shown to be two of 57 (3·5%) compared with 0 of 46 for those seated elsewhere, with only the rear section of the plane studied.^9^ Following the study of two long-haul flights, Foxwell *et al*.^6^ suggested that the risk of contracting influenza A(H1N1)pdm09 from fellow passengers was increased by 1·4% (95% CI 0·5–3·4%) in those seated within two rows of an infectious case, but the overall response rate in this study was low at 43%, raising the possibility of selection bias. An investigation of two flights landing in North America, of between 1·5- and 2·5-hour duration, demonstrated a risk ratio of 3·0 (95% CI 0·7–12·7) for those seated within two rows of an infectious case of influenza A(H1N1)pdm09 compared with those who were not.^16^

The results of laboratory testing in this present study are largely consistent with the clinical case definitions. All four *infected in-flight* cases tested using PCR were positive, suggesting specificity of the CDC clinical case definition; only one of four *infectious* cases tested was positive, but this is unsurprising given the length of time (5–15 days) between symptom onset in Mexico and return to the UK. Of the four *infected in-flight* cases confirmed using PCR testing, two of four (50%) cases were seated within two rows of an *infectious* case (RR 0·7; 95% CI 0·10–4·7). Serological testing was only available for two cases (both *infected in-flight*) with ILI and was negative in one case with a previously positive PCR, perhaps as a result of early timing of the test or a false-negative result following administration of oseltamivir. Serological data were considered in the light of potential cross-reactivity of antibody from seasonal influenza A(H1N1)^15^; antibody titres ≥1:32 by HI were only considered ‘suggestive’, not as confirmation of previous infection. Furthermore, serological data were only interpreted in the context with additional information, namely PCR and ILI data.

Influenza transmission in-flight can occur via several pathways: direct physical contact, fomites, direct droplet spread and suspended small particles.^17^ Aircraft cabins contain air that is recycled through high-efficiency particulate air (HEPA) filtration via a laminar flow pattern, reducing longitudinal spread fore and aft of the vessel.^18^ The risk of airborne transmission of pathogens is therefore likely to be greatest in adjacent rows, although computational studies have demonstrated spread of droplets up to seven rows within 4 minutes^19^; also perturbations of airflow are likely to occur as passengers walk through the cabin.^20^ Influenza virus-containing aerosol shedding can occur during normal tidal breathing, leading to suspension of particles in ambient air and potential disease transmission during air travel.^21^,^22^ In one study, 70% of exhaled aerosol particles in subjects with influenza were between 0·3 μm and 0·5 μm,^23^ therefore largely removed if passed through HEPA filtration.^24^ Low absolute humidity on aircraft has the theoretical potential to increase influenza survival and transmission.^17^ Using quantitative microbial risk assessment, Wagner *et al*.^25^ demonstrated that the risk of contracting influenza A(H1N1)pdm09 is unlikely to extend beyond each individual cabin and is lower in first compared with economy class; their estimates of 5–10 new infections from a single case over an 11-hour flight are consistent with the findings of this present study.

### Limitations

Limitations of this study include selection and measurement biases, limited sample size and the potential for influenza A(H1N1)pdm09 transmission beyond the flight.

Information sufficient to determine case status was available for 239 of 278 (86·0%) passengers on board the flight, with more detailed clinical data obtained from 224 of 278 (80·6%) passengers, the high response reducing the potential effect of selection bias. No data are available regarding the demographics or case status of non-responders to the cohort study and contact tracing exercise, unless recorded during enhanced surveillance. However, there is no suggestion that this group were aware of, and therefore could be selected by, the exposure studied. The majority of passengers with unknown case status belonged to groups of four to six travellers, where only contact details of the lead passenger were available, further suggesting that non-response was unrelated to individual case status or exposure, and therefore unlikely to bias the results of this study. The use of a clinical case definition has the potential to result in incorrect classification of case status, thereby under- or overestimating the attack rate. The specificity of 100% for the four *infected in-flight* cases tested with PCR provides some validation to the CDC case definition chosen. The validity of defining true *infectious* cases was potentially reduced by the low PCR positivity rate (1 of 4; 25%) and lack of serological testing among this group. The potential for observer bias was minimised as interviewers would not be aware of the location of infectious cases in the cohort study, but recall bias among respondents aware of the reported cases remains a possibility.

Despite the large number of passengers on the flight, the low attack rate reduces the power in this study to test the primary hypothesis, the AR in those seated or not seated within two rows of an infectious case was almost identical, but a much larger study, in practical terms a meta-analysis of several flights, would be required to test the hypothesis with statistical integrity.

In calculating the AR, the presumption is made that all newly infected cases contracted influenza A(H1N1)pdm09 during the course of the flight, but this requires the validity of several assumptions. The incubation period used in this study was 0–6 days, estimated from modelling studies among the UK population following the pandemic.^13^ A systematic review of influenza A suggested an incubation period of 0·7–2·8 days, but a longer time period in this study was used because it is influenza A(H1N1)pdm09 specific and onset of disease up to 6 days after the flight was considered more likely to be from passenger contacts than elsewhere.^26^ Reported median incubation periods for influenza A(H1N1)pdm09 include 0·8, 2·1, 3 and 4·3 days, all consistent with the pattern of the epidemic curve in this present study.^13^,^27^–^29^

Despite the first reported cases of influenza A(H1N1)pdm09 arriving on the flight studied, there is evidence to suggest circulating disease in the UK prior to this. A phylogenetic study estimated from divergence that two of 13 strains of influenza A(H1N1)pdm09 may have been circulating up to 8 days before the arrival of the flight, although the risk of transmission to the passengers studied remains unknown.^30^

Exhaustive attempts were made in the cohort study to identify common links, such as accommodation and excursions in Mexico, family groups and toilet use on board. No clear links were identified apart from that between *infectious* case 5 and *infected in-flight* case 13, but the potential for becoming infected prior to the flight in Mexico remains for all infected passengers, and therefore the AR may be an overestimate.

Other potential opportunities for infection include via aerosol spread while travelling in close proximity to an infectious case on a low-humidity air-conditioned charter bus to the airport and via direct contact in a potentially crowded departure lounge.

At the time of the cohort study, the aircraft toilet locations were not known to health protection staff, and there is likely to be considerable non-differential misclassification in self-reported use. Therefore, any increased risk of illness, if present, associated with using the same toilet as an *infectious* case was unlikely to be identified by this study. The onset of symptoms 5–15 days prior to the flight for *infectious* cases raises the possibility of resort exposure leading to the infection of those *infected in-flight* and demonstrates the additional possible exposure to circulating disease in Mexico at the time of the flight. The shape of the epidemic curve, albeit for a small number of cases, does suggest increased transmission around the time of the flight.

### Implications for public health policy

The findings of this study do not support current WHO guidance regarding the contact tracing of passengers seated within two rows of an infectious case of influenza A(H1N1)pdm09. The complex transmission dynamics of influenza, including airborne, direct and surface transmission, make clear identification of those at risk and decisions about subsequent contact tracing challenging.^30^ Further cohort studies after the identification of infectious passengers, followed by meta-analysis of data, are required to validate or refine current guidance.
